# The Correlation of Enhanced Osteogenic Capability in Titanium Alloy Implants Through Modulation of the Runx2/BSP Pathway

**DOI:** 10.1111/jcmm.71254

**Published:** 2026-07-09

**Authors:** Zhuo Wang, Huayan Guo

**Affiliations:** ^1^ Department of Stomatology Shanghai East Hospital, School of Medicine, Tongji University Shanghai China

**Keywords:** alloys, biocompatible materials, bone sialoprotein, osteogenesis, Runx2 transcription factor, titanium

## Abstract

We investigated the mechanical properties, biocompatibility and osteogenic potential of titanium alloy implants (Ti6Al4V group) compared to conventional alloy implants (Ti4Al2V group). The mechanical performance tests, including ultimate tensile strength, elongation at break, yield strength and ultimate compressive strength, demonstrated superior results for the titanium alloy implants. In vitro cell experiments, comprising CCK‐8 assay and ALP (alkaline phosphatase) staining, revealed significantly higher cell viability and osteogenic activity in the Ti6Al4V group. Additionally, in vivo animal experiments, including HE staining, flow cytometry, immunofluorescence assay. Ti‐6Al‐4V implants exhibited superior mechanical properties compared with conventional alloy implants, with a higher ultimate tensile strength (1200 ± 50 vs. 950 ± 45 MPa), greater elongation at break (15.2% ± 2.1% vs. 9.8% ± 1.7%), higher compressive yield strength (850 ± 40 vs. 720 ± 35 MPa) and higher ultimate compressive strength (1300 ± 60 vs. 1100 ± 50 MPa) (all *p* < 0.05). The elastic modulus of Ti‐6Al‐4V (10.8 ± 0.15 GPa) was lower than that of the conventional alloy (16.2 ± 0.18 GPa), closer to cancellous bone (~4 GPa), suggesting reduced stress shielding. In vitro, Ti‐6Al‐4V implants supported significantly higher MC3T3‐E1 proliferation at 48 h (*p* < 0.01) and enhanced osteogenic differentiation, with ALP staining intensity of 450 ± 35 vs. 320 ± 28 and a higher percentage of ALP‐positive cells (85% ± 4% vs. 60% ± 5%) compared with controls (both *p* < 0.05). In vivo, H&E staining showed more abundant and better‐organised bone trabeculae around Ti‐6Al‐4V implants, while flow cytometry revealed reduced apoptosis in peri‐implant tissues (*p* < 0.05). At the molecular level, Ti‐6Al‐4V implants significantly upregulated osteogenic markers: Runx2 protein expression was increased by 33% and BSP by 42% relative to the control alloy, accompanied by higher levels of osteocalcin (OCN) and collagen type I alpha 1 (COL1A1) (all *p* < 0.05). Ti4Al2V groupTi4Al2V group. These findings suggest that titanium alloy implants exhibit better biocompatibility and osteogenic capacity than conventional alloy implants, providing a strong foundation for their potential application in clinical settings.

## 1 Introduction

Tooth loss is a common clinical problem that affects millions of individuals worldwide and is associated with impaired mastication, speech difficulties and compromised facial aesthetics [1, 2]. Dental implants have become the preferred treatment option for replacing missing teeth, providing a stable and long‐lasting alternative to conventional removable dentures and fixed bridges [3]. These implants are surgically inserted into the jawbone and function as artificial tooth roots, offering reliable support for prosthetic restorations such as crowns, bridges or overdentures [4]. The long‐term success of dental implants critically depends on osseointegration, that is, the direct structural and functional connection between bone and the implant surface [5]. However, achieving rapid and stable osseointegration, particularly in patients with compromised bone quality, remains a significant clinical challenge and continues to drive research into improved implant materials.

Titanium and its alloys are widely used as implant materials because of their excellent mechanical strength, corrosion resistance and biocompatibility [6]. Among them, Ti‐6Al‐4V has become one of the most commonly used alloys in both orthopaedic and dental applications. Numerous studies have reported that Ti‐6Al‐4V exhibits favourable osseointegration and can promote bone formation at the implant–bone interface [7]. Nevertheless, most previous investigations have either evaluated Ti‐6Al‐4V in isolation, compared it with commercially pure titanium, or focused primarily on surface modifications. As a result, the specific contribution of alloy composition and mechanical properties per se to cellular behaviour and bone integration, especially when comparing closely related Ti–Al–V formulations under matched surface conditions, remains insufficiently understood.

At the molecular level, the Runx2/bone sialoprotein (BSP) pathway has emerged as a key regulator of bone development and regeneration [8, 9]. Runx2 is a master transcription factor that governs osteoblast differentiation [10], whereas BSP is an essential bone matrix protein involved in cell adhesion, mineralisation and matrix maturation. Modulation of the Runx2/BSP axis has the potential to enhance osteoblast activity, promote bone mineral deposition and accelerate implant integration with surrounding bone tissue [11, 12]. Although previous studies have examined osteogenic responses to titanium and its alloys, detailed in vivo evidence linking specific titanium alloy compositions to activation of the Runx2/BSP pathway and downstream matrix proteins in a dental implant context is still limited.

In this study, we specifically investigate the mechanical properties and osteogenic potential of two closely related titanium alloys used as dental implants: Ti‐6Al‐4V (Ti6Al4V group; 90.2% Ti, 5.8% Al, 4.0% V) and a conventional Ti–Al–V alloy with lower Al and V content (Ti4Al2V group; 93.7% Ti, 4.1% Al, 2.2% V). Importantly, the two implant types were manufactured with comparable surface topography and micro‐roughness, as confirmed by scanning electron microscopy and energy‐dispersive X‐ray spectroscopy, allowing us to minimise the influence of surface morphology and isolate the effects of alloy composition and mechanical behaviour on biological outcomes. We combined quantitative mechanical testing (tensile strength, elongation, compressive strength, elastic modulus), in vitro assessment of cell proliferation and early osteogenic differentiation (CCK‐8, ALP) and in vivo evaluation in a rat mandibular implant model, including histology, apoptosis analysis and molecular profiling of osteogenic markers.

By integrating these multi‐level analyses, our work extends existing research on Ti‐6Al‐4V implants in both orthopaedic and dental fields in two important ways. First, we provide a direct, controlled comparison between Ti‐6Al‐4V and a conventional Ti–Al–V alloy of lower Al/V content under matched surface conditions, thereby clarifying how subtle differences in alloy composition and mechanical properties translate into changes in osteoblast activity and bone integration. Second, we demonstrate that the superior osteogenic performance of Ti‐6Al‐4V is associated with upregulation of the Runx2/BSP axis and downstream markers such as osteocalcin (OCN) and collagen Type I alpha 1 (COL1A1) in peri‐implant bone. These findings offer new mechanistic insight into how titanium alloy composition can be optimised to simultaneously improve mechanical performance and biological integration in dental implant applications, with direct relevance for the design of implants for patients with compromised bone conditions.

## 2 Materials and Methods

### 2.1 Mechanical Performance Testing

Mechanical performance testing was conducted to compare the mechanical properties of the titanium alloy implants (Ti6Al4V group, *n* = 3) and conventional alloy implants (Ti4Al2V group, *n* = 3). Two key tests, tensile testing and compression testing, were performed to assess the materials' mechanical behaviour under different loading conditions.

#### 2.1.1 Tensile Testing

For each alloy, *n* = 3 specimens were prepared for tensile testing and *n* = 5 for compression testing. The tensile strength and elongation properties of the implants were evaluated using a universal testing machine (WDW‐Y, Shanghai Shengshi Huike Testing Equipment Co. Ltd.). Specimens were prepared according to the ASTM E8 standard for tensile testing of metallic materials. The test was conducted at a strain rate of 0.005 s^−1^, and the specimens were subjected to uniaxial tension until failure. Tensile strength (MPa) and elongation at break (%) were recorded to assess the mechanical performance of the titanium alloy implants in comparison to the Ti4Al2V group. All tests were performed at room temperature (22°C ± 1°C), and each test was repeated three times to ensure reproducibility. The data were analysed statistically to assess any significant differences between the experimental and Ti4Al2V groups.

#### 2.1.2 Compression Testing

For each alloy, *n* = 3 specimens were prepared for tensile testing and *n* = 5 for compression testing. Compression testing was carried out to determine the mechanical properties of the titanium alloys under compressive conditions. The tests followed the ASTM E9 standard, with specimens machined to the recommended dimensions. Compressive yield strength (MPa) and ultimate compressive strength (MPa) were measured using a compression testing machine (SHK‐A101, Suzhou Jianzhuo Instrument Technology Co. Ltd.). The test was performed at a constant strain rate of 0.002 s^−1^ until specimen failure or deformation. Results were used to evaluate the compressive resistance of the titanium alloy implants compared to the conventional alloy implants. All tests were performed at room temperature (22°C ± 1°C), and each test was repeated three times to ensure reproducibility. The data were analysed statistically to assess any significant differences between the experimental and Ti4Al2V groups.

### 2.2 Cell Culture and Treatment

The cell line MC3T3‐E1 was sourced from Wuhan Punuo Life Technology Co. Ltd. and cultured in DMEM medium (Thermo Fisher, USA) supplemented with 1% penicillin/streptomycin and 10% FBS (Biologic Industries), maintaining a temperature of 37°C with 5% CO_2_ in a humidified environment. The cells were cultured in DMEM supplemented with penicillin/streptomycin and FBS, which are commonly used to support cell growth and provide essential nutrients. To investigate the role of titanium alloy implants in enhancing osteogenic capability, the study was divided into two groups: Ti4Al2V group: Cells were seeded on Conventional Alloy Implants. Ti6Al4V group: Cells were seeded on Titanium Alloy Implants. Approximately 1 × 10^4^ MC3T3‐E1 cells were seeded onto each implant type in 24‐well plates, with triplicates set up for each group to ensure statistical reliability. The cells were incubated under standard conditions (37°C, 5% CO_2_) for the duration of the study, allowing them to attach to the implant surfaces.

### 2.3 CCK‐8 Assay

The CCK‐8 assay was employed to evaluate the proliferation rate of MC3T3‐E1 cells seeded on the implant materials. Approximately 1 × 10^4^ cells per well were seeded onto 24‐well plates containing the implant materials (either Conventional Alloy Implants or Titanium Alloy Implants). Each condition was set up in triplicates to ensure reproducibility. The cells were incubated at 37°C with 5% CO_2_ in a humidified environment for 24 h, allowing them to attach and proliferate. 10 μL of the CCK‐8 reagent was added directly to each well containing 100 μL of culture medium. The plates were then incubated for an additional 2 h at 37°C to allow the formation of a water‐soluble formazan dye by viable cells. After the incubation, the absorbance at 450 nm was measured using a microplate reader. The optical density (OD) values were used to determine the relative number of viable cells in each well.

### 2.4 ALP Staining Assay

ALP staining assay was utilised to assess osteoblast activity in MC3T3‐E1 cells cultured on the implant materials. Cells were stained using a commercial ALP staining kit (Beyotime Biotechnology) according to the manufacturer's protocol. The cells were incubated in the ALP staining solution for 30 min at 37°C. During this incubation, ALP activity catalyses the conversion of the substrate to a coloured product, marking regions of high osteoblast activity. After staining, the wells were gently rinsed with distilled water to remove excess staining solution. ALP‐positive areas (indicating osteoblast activity) were visualised under a light microscope, and images were captured to document the results. The stained areas were dissolved in a suitable solvent, and the optical density (OD) was measured at 405 nm using a microplate reader. The results were expressed as ALP activity relative to total protein content, determined via a separate protein quantification assay.

### 2.5 In Vivo Animal Experiment

To evaluate the osseointegration performance and tissue response of Ti6Al4V in comparison to conventional alloy implants (Ti4Al2V group), an in vivo rat model was used. This study followed all applicable institutional and national guidelines for the care and use of animals, with approval from the Animal Ethics Committee (Protocol No. AEC‐2023‐045). Cylindrical, screw‐type implants were manufactured from Ti‐6Al‐4V (Ti6Al4V group) and a conventional Ti–Al–V alloy (Ti4Al2V group). All implants had identical macroscopic geometry, with a diameter of 2.0 mm and a length of 4.0 mm, a thread pitch of 0.6 mm and a total threaded length of 3.5 mm. The implant surface was machined to obtain a comparable micro‐roughness between the two alloys, as confirmed by SEM and profilometry.

#### 2.5.1 Animal Model and Grouping

Sixteen male Sprague–Dawley rats (weight: 250–300 g, age: 8–10 weeks) were used in the study. The animals were randomly divided into two groups (*n* = 8 per group): Ti4Al2V group: Conventional alloy implants; Ti6Al4V group: Titanium alloy implants. Each rat received one implant in each hemimandible (two implants per animal), resulting in a total of 16 implants per group.

#### 2.5.2 Surgical Procedure

Before surgery, the rats were anaesthetised with intraperitoneal injections of ketamine (80 mg/kg) and xylazine (10 mg/kg). The surgical site (mandible) was shaved and disinfected using povidone‐iodine solution. A 1.5 cm incision was made along the mandible, and the periosteum was carefully elevated to expose the bone. A dental drill was used to create a standardised defect at the mandibular site. The implants were then inserted into the prepared bone defect according to their respective groups. Following implantation, the surgical site was closed with absorbable sutures. Postoperative analgesia was provided with buprenorphine (0.05 mg/kg, subcutaneously) every 12 h for 48 h.

#### 2.5.3 Postoperative Care and Euthanasia

The animals were housed in individual cages under a 12‐h light/dark cycle, with access to standard chow and water ad libitum. They were monitored daily for signs of infection, wound healing and general health. After 8 weeks, the rats were euthanised via CO_2_ inhalation followed by cervical dislocation, and the mandibles were harvested for analysis.

### 2.6 Evaluation of Osseointegration and Tissue Response

Undecalcified sections of the mandibles were prepared, and histological analysis was conducted using haematoxylin and eosin (H&E) staining to evaluate the tissue response and the extent of osseointegration. Histomorphometric analysis was carried out to quantify the bone‐to‐implant contact (BIC) percentage, providing a comparison between the control and Ti6Al4V groups.

### 2.7 Flow Cytometry Assay

The mandible tissues were subjected to flow cytometry analysis following the manufacturer's instructions. After collection, the tissues were stained with Annexin V‐FITC and Propidium Iodide (PI) in a dark environment. Subsequently, apoptosis levels in all groups were quantified using a flow cytometer.

### 2.8 Immunofluorescence Assay

Begin the immunofluorescence assay by applying stabilisation and infiltration techniques to mandible tissues. Next, immerse the tissues in a bath containing primary antibodies. After allowing sufficient time for incubation with the primary antibodies, rinse the samples promptly to eliminate any unbound antibodies. Then, expose the mandible tissues' cells to a secondary incubation with fluorescent secondary antibodies, followed by another wash to remove any remaining unbound antibodies. Conclude the procedure by examining the treated samples using a fluorescence microscope for analysis.

### 2.9 Western Blotting

Protein extracts from the mandibles' tissues cells were resolved using 10% SDS‐PAGE and then transferred onto PVDF membranes. The membranes underwent a rinse with TBST to eliminate any non‐specific bindings. The primary antibodies used were β‐actin (1:1500, ab8226, Abcam), Runx2 (1:1000, #12556, CST) and BSP (1:500, ab307994, Abcam) to target the specific proteins and actin, followed by an overnight incubation at 4°C. Post‐incubation, TBST was used to wash away any non‐specifically bound primary antibodies. Subsequently, secondary antibodies from the same supplier were applied, and the membranes were incubated at room temperature for 2 hours. Afterward, the membranes were washed again with TBST to remove any residual antibodies. Detection of protein bands was carried out using an ECL chemiluminescence reagent and analysed accordingly.

### 2.10 qRT‐PCR

Total RNA was isolated from the mandibles tissues cells using the TRIzol Reagent (Beyotime, Shanghai) following the manufacturer's instructions. The mRNA was then converted into cDNA utilising the mRNA Reverse‐Transcription Kit (Beyotime, Shanghai). To quantify mRNA expression, quantitative PCR was conducted with the SYBR Green PCR Mix (Vazyme Biotech, Shanghai) on a Real‐Time PCR System. The relative expression levels were determined using the 2^−ΔΔ*Ct*^ method, with normalisation to GAPDH. The primers used were as follows: for Runx2, Forward: 5′‐ACTTCGTCAGCGTCCTATC‐3′ and Reverse: 5′‐CATCAGCGTCAACACCATC‐3′; for BSP, Forward: 5′‐TGCAGAGGACGTTCGGAAG‐3′ and Reverse: 5′‐CGAGAGTAGACACCGGGAAAG‐3′; and for GAPDH, Forward: 5′‐GGAGTCTACTGGCGTCTTCAC‐3′ and Reverse: 5′‐ATGAGCCCTTCCACGATGC‐3′.

### 2.11 Statistical Analysis

Statistical analyses were performed using Prism 8 software. The data were presented as the mean ± SD, with each experiment being repeated a minimum of three times. To determine differences between two groups, a *t*‐test was utilised. For comparisons among three or more groups, a one‐way ANOVA was employed. A *p*‐value less than 0.05 was deemed statistically significant.

## 3 Results

### 3.1 Material Characterisation and Elemental Composition Analysis of Titanium Alloy Implants

**FIGURE 1:**
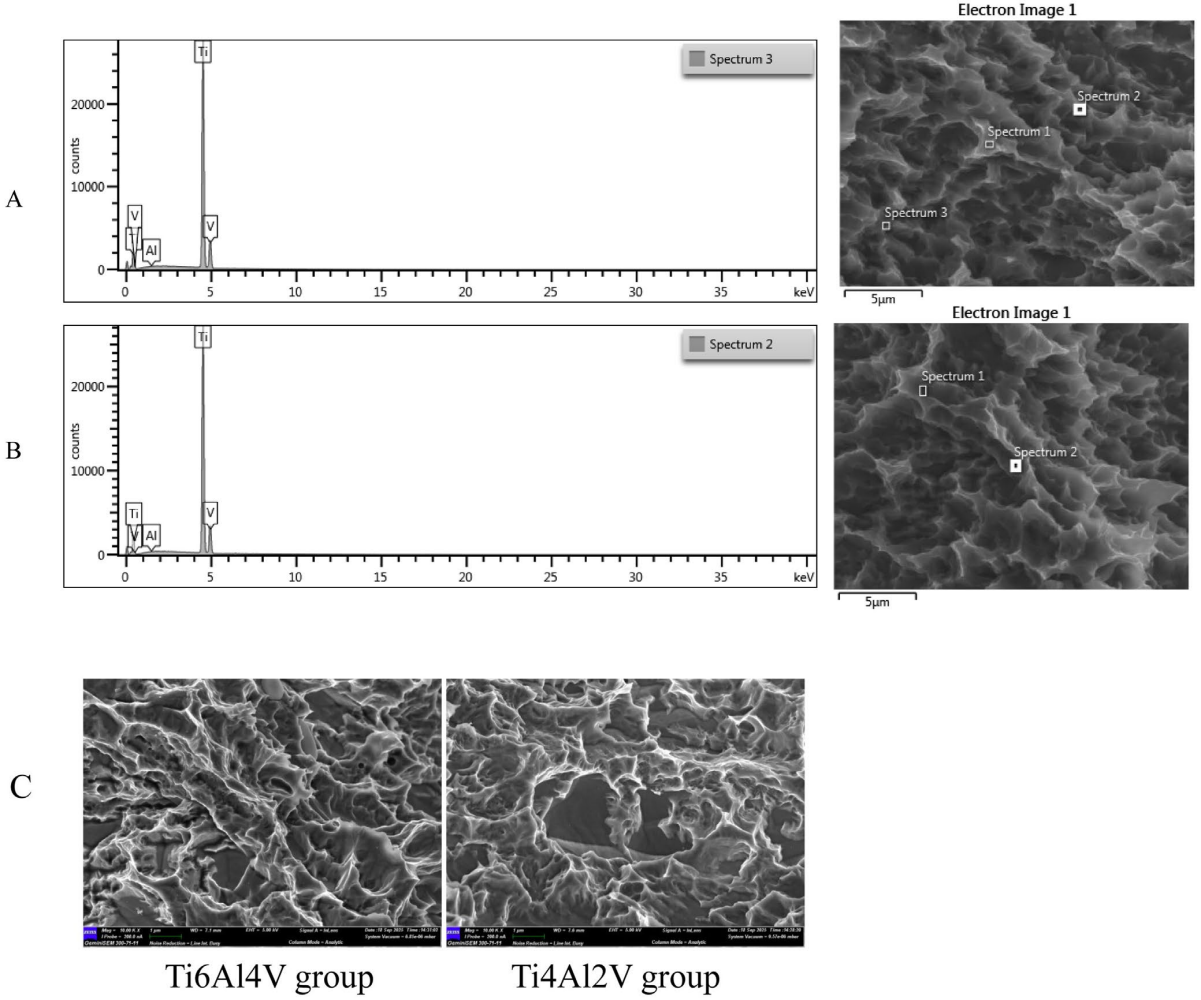
Energy‐Dispersive X‐ray Spectroscopy (EDS) spectra showing elemental peaks for both groups. (A) Actual photographs of Ti6Al4V group, (B) Actual photographs of Ti4Al2V group, (C) SEM images showing comparable surface morphologies of both implant groups.

Energy‐Dispersive X‐ray Spectroscopy (EDS) analysis confirmed the elemental composition of both implant groups (Figure 1A,B). The Ti6Al4V group (Ti‐6Al‐4V) showed mass percentages of 90.2% titanium, 5.8% aluminium and 4.0% vanadium, closely aligning with the theoretical composition. The Ti4Al2V group (conventional titanium alloy) exhibited 93.7% titanium, 4.1% aluminium and 2.2% vanadium. Both groups demonstrated high material purity with minimal trace elements detected. EDS mapping revealed a homogeneous distribution of alloying elements across the implant surfaces, confirming consistent material properties for a reliable experimental comparison between the two titanium alloy formulations. Scanning Electron Microscopy (SEM) analysis indicated comparable surface morphologies between both implant groups (Figure 1C). The Ti6Al4V group (Ti‐6Al‐4V) and Ti4Al2V group (conventional titanium alloy) displayed similar surface topographies with uniform micro‐roughness patterns and consistent surface textures. Both groups exhibited typical machined surface characteristics with parallel grooves and minimal surface defects. The surface roughness parameters and topographical features were homogeneous across both groups, ensuring equivalent initial surface conditions for subsequent biological response evaluations.

### 3.2 Mechanical Performance Testing Results

Mechanical testing revealed that the Ti6Al4V group exhibited superior tensile and compressive properties compared with the conventional Ti–Al–V alloy implants (Ti4Al2V group). For each alloy, *n* = 3 specimens were tested, and values are presented as mean ± SD.

**FIGURE 2:**
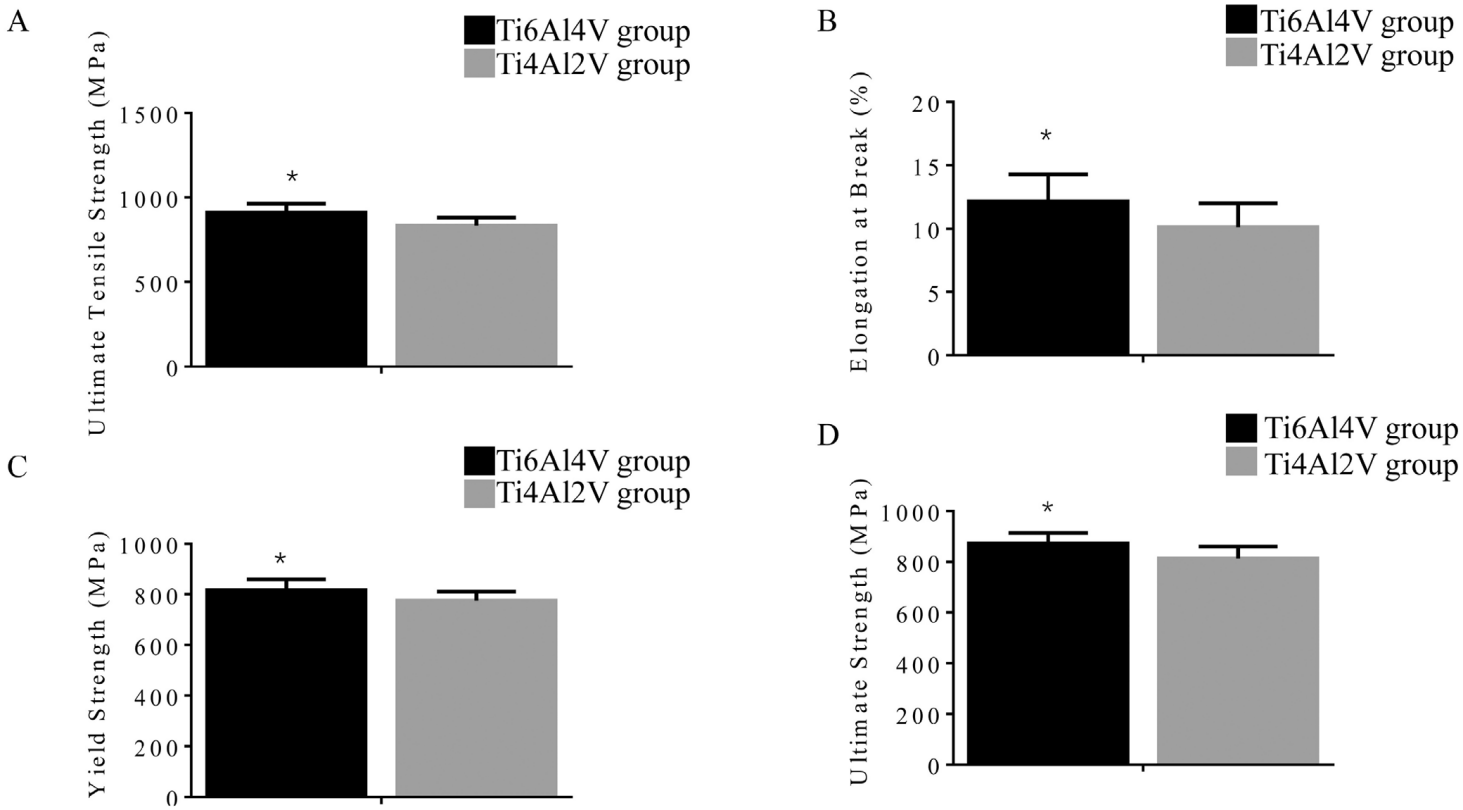
Mechanical properties of titanium alloy implants and conventional alloy implants. (A) Tensile strength; (B) Elongation at break; (C) Yield strength; (D) Ultimate compressive strength. Compared with the Ti4Al2V group, *p < 0.05.

In tension, the ultimate tensile strength (UTS) of the Ti‐6Al‐4V implants was significantly higher than that of the conventional alloy (1200 ± 50 MPa vs. 950 ± 45 MPa, *p* < 0.05; Figure 2A). The elongation at break was also markedly increased in the Ti6Al4V group (15.2% ± 2.1% vs. 9.8% ± 1.7%, *p* < 0.05; Figure 2B), indicating enhanced ductility and a greater ability to undergo plastic deformation before failure.

Under compressive loading, Ti‐6Al‐4V implants demonstrated significantly greater strength than the control alloy. The compressive yield strength reached 850 ± 40 MPa for the Ti6Al4V group compared with 720 ± 35 MPa for the Ti4Al2V group (*p* < 0.05; Figure 2C). Similarly, the ultimate compressive strength was higher in Ti‐6Al‐4V implants (1300 ± 60 MPa) than in the conventional alloy implants (1100 ± 50 MPa, *p* < 0.05; Figure 2D). These findings indicate that the experimental alloy is more resistant to deformation and failure under both tensile and compressive forces.

Static compression testing further showed a substantial difference in elastic modulus between the two alloys. Ti‐6Al‐4V exhibited an elastic modulus of 10.8 ± 0.15 GPa, whereas the conventional alloy showed a higher modulus of 16.2 ± 0.18 GPa. The lower modulus of Ti‐6Al‐4V is closer to that of cancellous bone (~4 GPa), suggesting a reduced risk of stress shielding and more favourable stress distribution at the implant–bone interface.

### 3.3 CCK‐8 Assay Results

**FIGURE 3:**
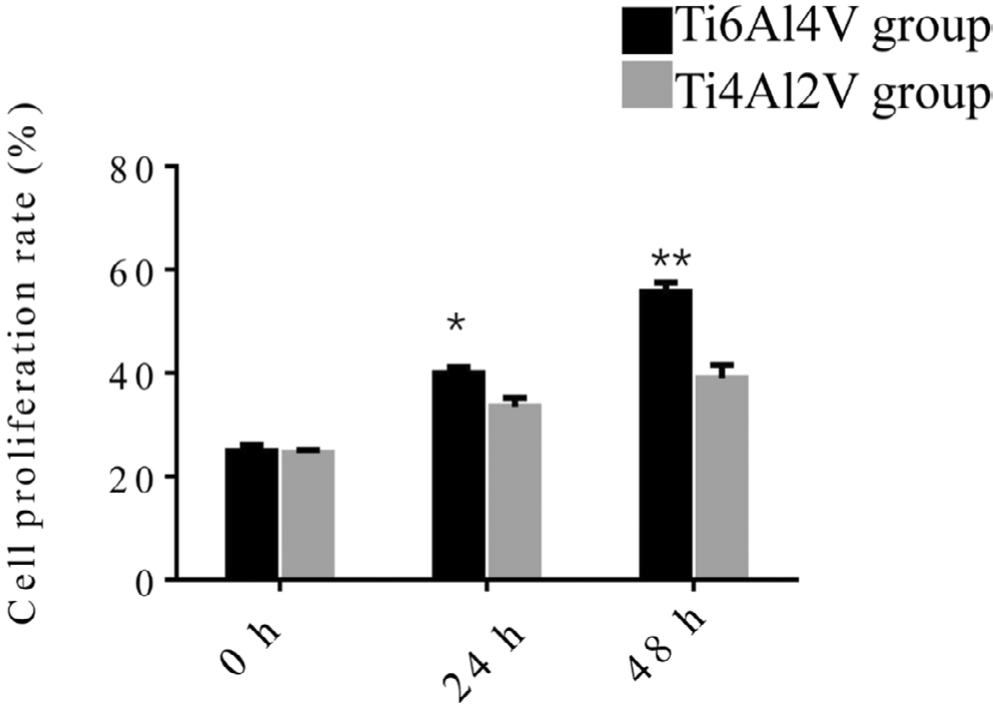
The CCK‐8 assay was used to evaluate the cell proliferation rate of MC3T3‐E1 cells on both Ti6Al4V group and Ti4Al2V group. Compared to the Ti4Al2V group, *p < 0.05 and **p < 0.01 indicate statistically significant differences.

The CCK‐8 assay was used to evaluate the proliferation of MC3T3‐E1 cells on both titanium alloy implants and conventional alloy implants. At the initial time point (0 h), there was no significant difference in cell proliferation between the experimental and Ti4Al2V groups (*p* > 0.05). This indicates that both groups had similar initial cell attachment rates, confirming equivalent seeding densities and starting conditions. At 24 h, the MC3T3‐E1 cell proliferation rate in the Ti6Al4V group was significantly higher than in the Ti4Al2V group (*p* < 0.05). This trend continued at 48 h, with the titanium alloy implants showing a significantly increased proliferation rate compared to the conventional alloy implants (*p* < 0.01), as shown in Figure 3. These results indicate that the titanium alloy implants promoted better cell growth over time.

### 3.4 ALP Staining Assay Results

**FIGURE 4:**
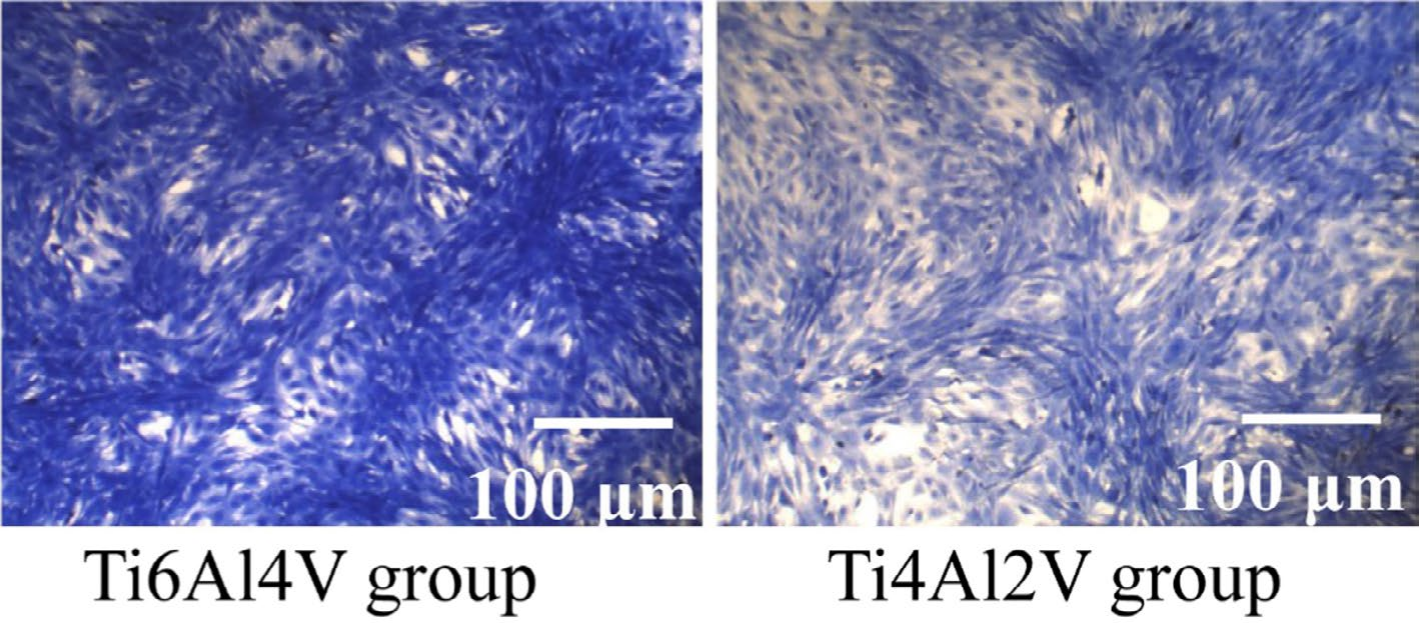
The ALP staining assay was used to evaluate the cell activity of MC3T3‐E1 cells on both Ti6Al4V group and Ti4Al2V group.

The ALP staining assay was performed to assess the cell activity of MC3T3‐E1 cells seeded on both titanium alloy implants and conventional alloy implants. After staining, the cells exhibited varying degrees of alkaline phosphatase (ALP) activity, a key indicator of osteoblast differentiation. The Ti6Al4V group showed significantly stronger ALP staining intensity compared to the Ti4Al2V group, indicating enhanced osteogenic activity. The staining intensity in the Ti6Al4V group was significantly higher (*p* < 0.05) than in the Ti4Al2V group, as shown in Figure 4, indicating increased cell activity and osteogenic potential on the titanium alloy surface.

The ALP staining assay was performed to assess the osteogenic activity of MC3T3‐E1 cells seeded on both Ti6Al4V group and Ti4Al2V group. The intensity of ALP staining, a key marker of osteoblast differentiation, was used to evaluate the degree of osteogenic activity. The cells on the titanium alloy implants exhibited significantly stronger ALP staining compared to those on the conventional alloy implants. The staining intensity in the Ti6Al4V group was quantified using ImageJ software, and the Ti6Al4V group showed an average ALP intensity score of 450 ± 35, while the Ti4Al2V group had a significantly lower ALP intensity score of 320 ± 28 (*p* < 0.05) (Figure 4). This difference indicates that the titanium alloy implants promote a higher level of osteogenic activity, as evidenced by the enhanced ALP expression on their surface. Additionally, the percentage of ALP‐positive cells was significantly greater in the Ti6Al4V group, with 85% ± 4% of cells staining positively for ALP, compared to 60% ± 5% of cells in the Ti4Al2V group (*p* < 0.05). This further supports the conclusion that the titanium alloy surface induces greater osteogenic potential, as it stimulates a higher proportion of osteoblasts to undergo differentiation. Overall, these findings confirm that the titanium alloy implants exhibit significantly enhanced osteogenic activity compared to the conventional alloy implants, suggesting their superior potential for supporting bone regeneration and integration.

### 3.5 HE Staining Results

**FIGURE 5:**
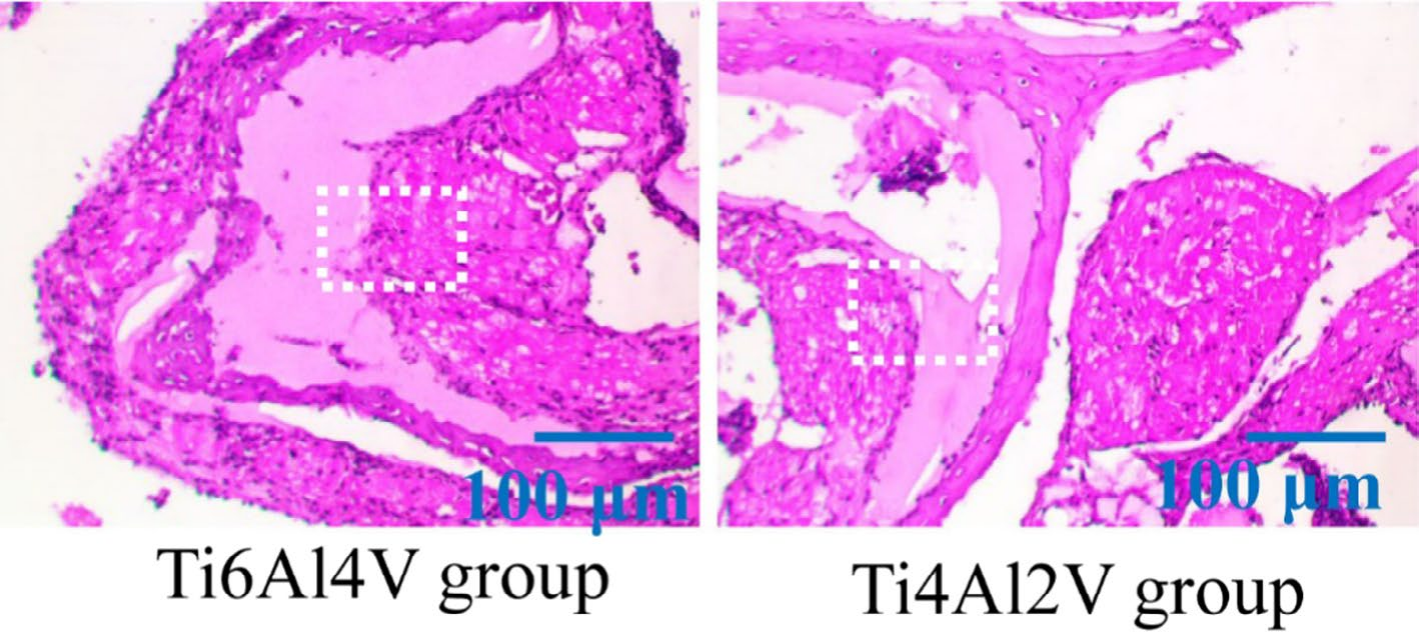
HE staining was utilised to examine the development and morphology of the mandibular bone tissue in SD rats around both titanium alloy and conventional alloy implants.

HE staining was performed to observe the growth and morphology of bone tissue surrounding the implants in both the titanium alloy implants and conventional alloy implants. The histological sections revealed clear differences in bone tissue growth between the two groups. In the Ti6Al4V group, the new bone tissue surrounding the titanium alloy implants appeared more abundant and well‐organised, with evidence of enhanced osseointegration. In contrast, the Ti4Al2V group showed less bone tissue formation, with less organised and less dense bone structures around the conventional alloy implants, as shown in Figure 5. The Ti6Al4V group exhibited significantly better bone tissue formation and organisation around the implant site. Thick layers of bone trabeculae were observed in close proximity to the titanium alloy implants, indicating strong osseointegration. In the Ti4Al2V group, bone growth was less pronounced, with thinner and less dense trabeculae observed surrounding the conventional alloy implants.

### 3.6 The Effect of Titanium Alloy Implants in Apoptosis

**FIGURE 6:**
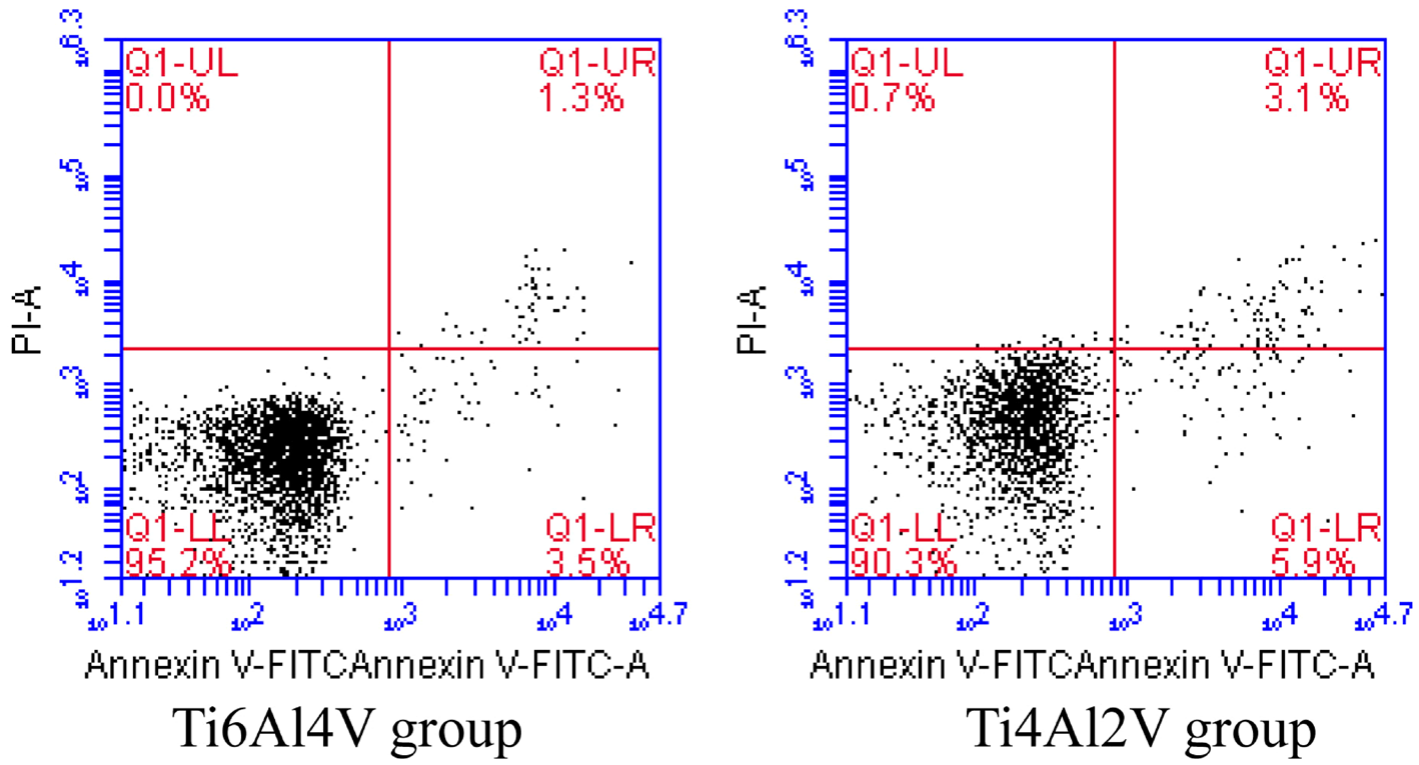
The apoptosis of bone tissue cells surrounding the implants in both the titanium alloy implants and conventional alloy implants were detected using the flow cytometry assay.

The flow cytometry assay was used to assess the apoptosis of bone tissue cells surrounding the implants in both the titanium alloy implants and conventional alloy implants. The flow cytometry results revealed a significantly lower percentage of apoptotic cells in the Ti6Al4V group compared to the Ti4Al2V group. The titanium alloy implants exhibited a reduced level of apoptosis, indicating better cell viability and biocompatibility of the titanium alloy compared to the conventional alloy implants. The percentage of early apoptotic cells in the Ti6Al4V group was significantly lower (*p* < 0.05) than in the Ti4Al2V group, suggesting enhanced cellular viability around the titanium alloy implants. The Ti6Al4V group also exhibited a lower percentage of late apoptotic cells compared to the Ti4Al2V group (*p* < 0.05), as shown in Figure 6, further confirming reduced cell death around the titanium alloy implants.

### 3.7 The Effect of Titanium Alloy Implants in Runx2

**FIGURE 7:**
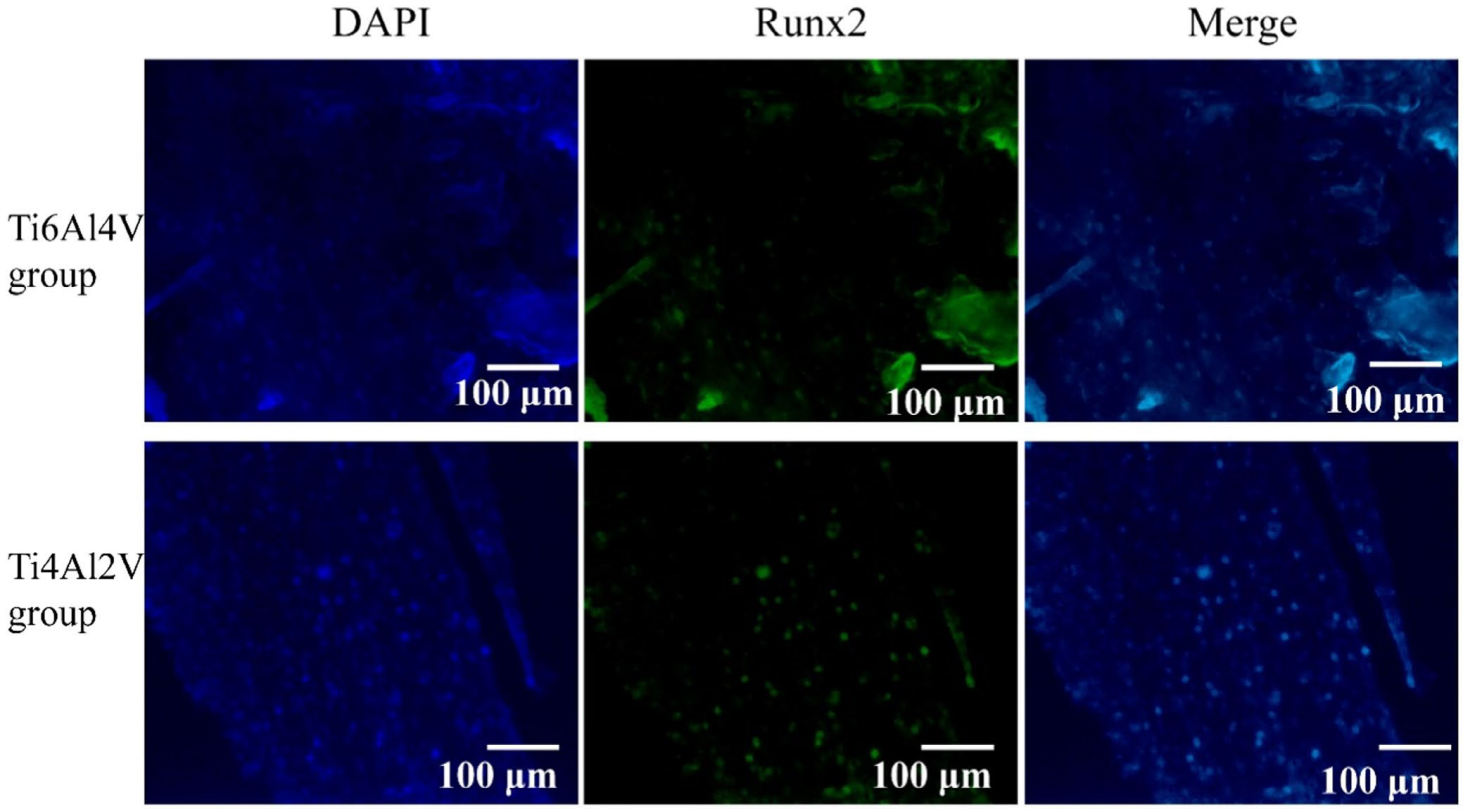
The expression levels of Runx2 of bone tissue cells surrounding the implants in both the titanium alloy implants and conventional alloy implants were detected using the immunofluorescence assay.

The immunofluorescence assay was used to assess the expression levels of Runx2, a key transcription factor involved in osteoblast differentiation, in the bone tissue cells surrounding the implants. The Ti6Al4V group was compared with the Ti4Al2V group to evaluate the osteogenic potential of the implant materials. The Ti6Al4V group exhibited significantly higher fluorescence intensity (*p* < 0.05), as shown in Figure 7, indicating enhanced osteogenic activity compared to the Ti4Al2V group. Runx2 Expression in Bone Tissue Cells The immunofluorescence images showed a notable difference in Runx2 expression between the two groups. The Ti6Al4V group demonstrated stronger fluorescence intensity, indicating higher levels of Runx2 expression in the bone tissue cells around the titanium alloy implants. In contrast, the Ti4Al2V group exhibited weaker fluorescence signals, suggesting lower Runx2 levels in the bone tissue cells surrounding the conventional alloy implants.

### 3.8 The Effect of Titanium Alloy Implants in Runx2 and BSP Proteins

**FIGURE 8:**
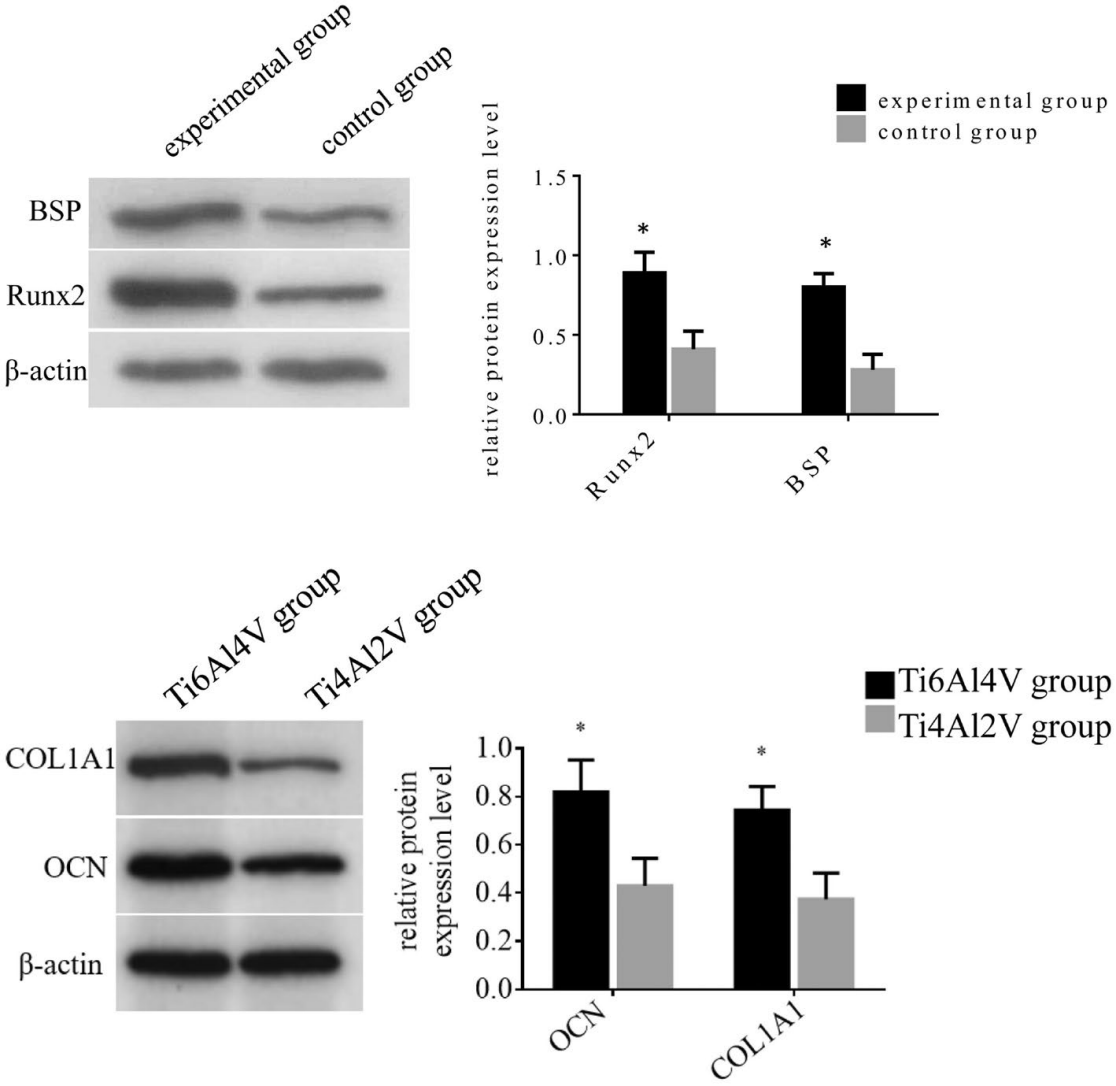
The expression levels of Runx2 and BSP proteins of bone tissue cells surrounding the implants in both the titanium alloy implants and conventional alloy implants were detected using the Western blot assay. Compared to the Ti4Al2V group, *p < 0.05 indicates statistically significant differences.

The Western blot analysis showed that the expression of Runx2 in the bone tissue cells surrounding the titanium alloy implants was significantly higher than in those surrounding the conventional alloy implants. This suggests that the titanium alloy implants promote greater osteogenic differentiation. The Ti6Al4V group exhibited a marked increase in Runx2 protein levels compared to the Ti4Al2V group, with band intensities significantly higher in the Ti6Al4V group (*p* < 0.05). Similarly, the expression of BSP, a marker of bone matrix mineralisation, was significantly higher in the Ti6Al4V group compared to the Ti4Al2V group. This indicates that the titanium alloy implants foster an improved environment for bone formation and mineralisation. The Ti6Al4V group showed significantly higher levels of BSP compared to the Ti4Al2V group (*p* < 0.05), as indicated by the increased band intensity on the Western blot, as shown in Figure 8. The WB results demonstrated that the expression levels of osteocalcin (OCN) and collagen Type I alpha 1 chain (COL1A1) proteins were significantly higher in the Ti6Al4V group compared to the Ti4Al2V group, with statistically significant differences observed. These results align with the enhanced osteogenic potential of titanium alloy implants, as indicated by other in vitro and in vivo assays. The increased expression of OCN and COL1A1 supports the notion that the experimental titanium alloy implants foster superior osteogenesis and bone tissue growth.

### 3.9 The Effect of Titanium Alloy Implants in Runx2 and BSP mRNA

**FIGURE 9:**
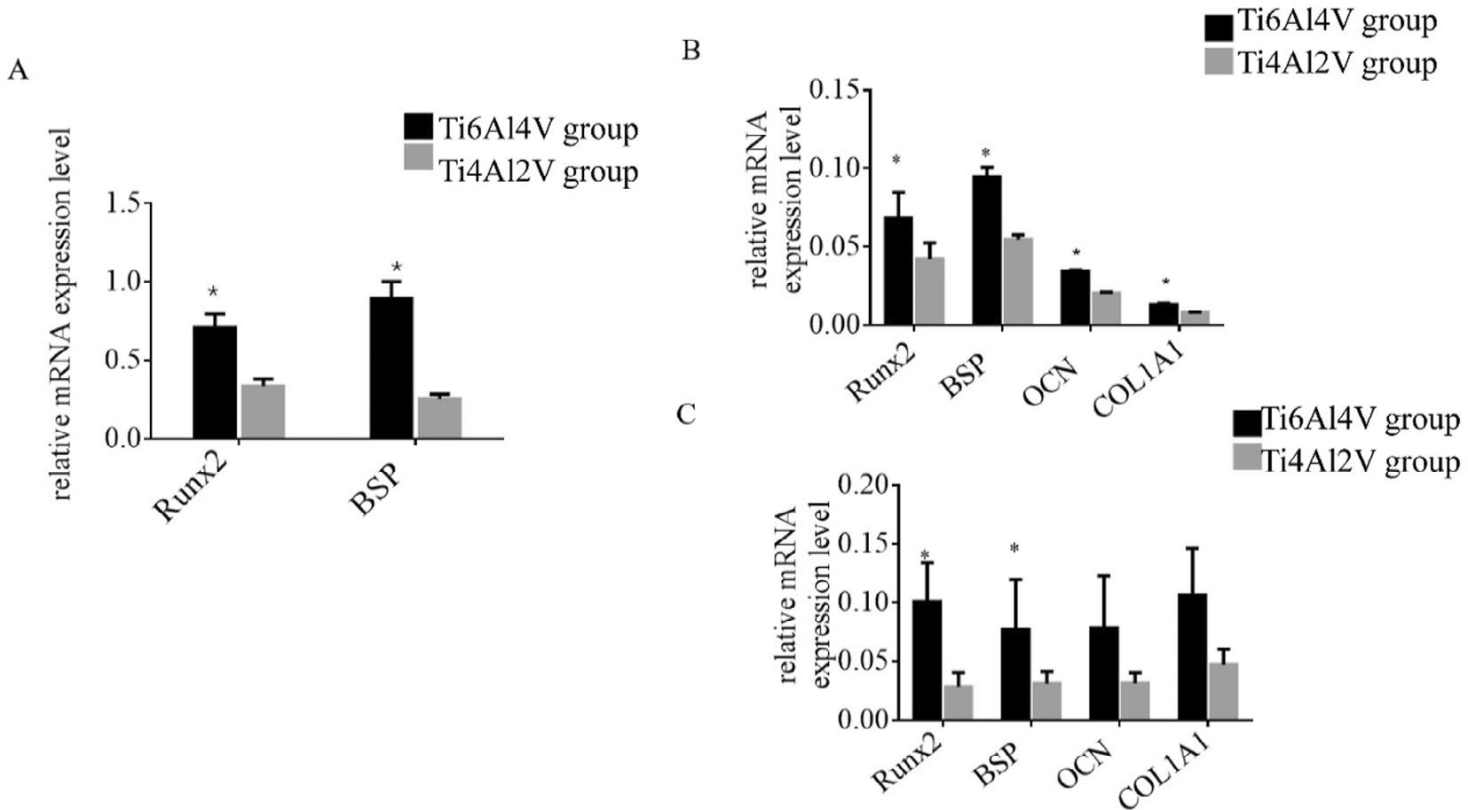
The expression levels of Runx2 and BSP mRNA in bone tissue cells surrounding the implants in both titanium alloy implants and conventional alloy implants were detected using qRT‐PCR. (A) Comparison of Runx2 and BSP mRNA expression levels in bone tissue cells surrounding Ti6Al4V and Ti4Al2V implants. (B) qRT‐PCR analysis of Runx2, BSP, OCN, and COL1A1 mRNA expression levels in bone tissue cells surrounding Ti6Al4V and Ti4Al2V implants after Runx2 knockdown. (C) qRT‐PCR analysis of Runx2, BSP, OCN, and COL1A1 mRNA expression levels in bone tissue cells surrounding Ti6Al4V and Ti4Al2V implants after BSP knockdown. Compared with the Ti4Al2V group, *p < 0.05 indicates statistically significant differences.

qRT‐PCR was performed to analyse the mRNA expression levels of Runx2 and bone sialoprotein (BSP) in bone tissue cells surrounding the implants. The comparison between the Ti6Al4V group and the Ti4Al2V group aimed to evaluate the osteogenic gene expression potential of each implant material. The qRT‐PCR analysis revealed that the mRNA expression of Runx2 in the Ti6Al4V group was significantly higher than in the Ti4Al2V group (*p* < 0.05, Figure 9A). This increase in Runx2 expression indicates enhanced osteogenic differentiation in the bone tissue cells surrounding the titanium alloy implants. Similarly, the mRNA expression of BSP, an essential marker for bone matrix mineralisation, was significantly higher in the Ti6Al4V group than in the Ti4Al2V group (*p* < 0.05). The qRT‐PCR results showed that after Runx2 knockdown, the mRNA expression levels of Runx2, BSP, OCN and COL1A1 were significantly higher in the Ti6Al4V group compared to the Ti4Al2V group, with statistically significant differences observed (Figure 9B). Similarly, after BSP knockdown, the mRNA expression levels of Runx2, BSP, OCN and COL1A1 were also significantly increased in the Ti6Al4V group compared to the Ti4Al2V group, with statistically significant differences (Figure 9C). These findings suggest that both Runx2 and BSP play a crucial role in regulating the osteogenic gene expression in the Ti6Al4V group.

## 4 Discussion

In this study, we systematically evaluated the mechanical properties, biocompatibility and osteogenic potential of titanium alloy implants compared to conventional alloy implants using a combination of in vitro and in vivo experiments. Our results demonstrate that titanium alloy implants significantly outperform conventional alloy implants in terms of mechanical strength, cell viability and osteogenic differentiation.

The mechanical performance data obtained in this study are consistent with, and in some aspects slightly exceed, previously reported values for Ti‐6Al‐4V used in orthopaedic and dental implants [13, 14]. Typical ultimate tensile strengths for Ti‐6Al‐4V alloys used in implantable devices have been reported in the range of approximately 900–1100 MPa, with elongation at break values of 10%–14% [15, 16]. In our study, the Ti‐6Al‐4V implants reached a UTS of 1200 ± 50 MPa and an elongation of 15.2% ± 2.1%, indicating that the material not only meets but slightly surpasses conventional performance benchmarks for clinical implant applications. Similarly, the compressive yield and ultimate strengths observed (850 ± 40 MPa and 1300 ± 60 MPa, respectively) fall within or above the ranges reported for load‐bearing Ti‐6Al‐4V components [15], suggesting that the implants can reliably withstand the complex tensile and compressive stresses encountered in the oral environment. Importantly, the elastic modulus of Ti‐6Al‐4V in our study (10.8 ± 0.15 GPa) is lower than that of the conventional Ti–Al–V alloy (16.2 ± 0.18 GPa) and closer to that of cancellous bone (~4 GPa). Previous reports have emphasised that a lower elastic modulus, while maintaining sufficient strength, is desirable to reduce stress shielding and promote more physiological load transfer to surrounding bone [17, 18]. Our findings therefore indicate that the Ti‐6Al‐4V implants not only satisfy established mechanical requirements for clinical use but also offer a more favourable stiffness profile that may support long‐term peri‐implant bone preservation.

The rationale for selecting MC3T3‐E1 pre‐osteoblasts as the in vitro model is that this murine cell line is one of the most widely used systems for evaluating osteogenic responses to biomaterials. MC3T3‐E1 cells exhibit well‐characterised osteoblastic differentiation, express classical markers such as ALP, Runx2 and BSP, and have been extensively employed to assess the osteoconductivity and biocompatibility of titanium and Ti‐6Al‐4V implants in previous studies [19, 20]. Using this standardised model allows our results to be directly compared with a large body of existing literature and provides a reproducible platform for testing how subtle differences in alloy composition influence early osteogenic events. However, the use of MC3T3‐E1 cells also introduces important limitations that should be acknowledged. As a murine pre‐osteoblast cell line, MC3T3‐E1 may not fully recapitulate the behaviour of human primary osteoblasts or mesenchymal stem cells, particularly with respect to species‐specific differences in signalling pathways and matrix production.

The in vivo animal experiments further confirmed the superior biocompatibility and osteogenic potential of Ti‐6Al‐4V implants. HE staining revealed more organised and abundant bone tissue formation around the Ti‐6Al‐4V implants compared with the conventional alloy. Flow cytometry showed a lower percentage of apoptotic cells in the Ti6Al4V group, indicating that Ti‐6Al‐4V implants support better cell survival in peri‐implant bone tissue [21, 22]. Immunofluorescence, Western blot and qRT‐PCR analyses all demonstrated significantly higher levels of osteogenic markers, including Runx2 and BSP, in the Ti‐6Al‐4V group [23]. These findings suggest that Ti‐6Al‐4V not only supports bone tissue growth but also actively promotes osteogenic differentiation at both the protein and mRNA levels. The increased expression of Runx2, a key transcription factor in osteoblast differentiation, and BSP, which is crucial for bone matrix mineralisation, further validates the osteoinductive properties of Ti‐6Al‐4V implants [24, 25].

Immunofluorescence, Western blot and qRT‐PCR analyses all demonstrated significantly higher levels of osteogenic markers, including Runx2 and BSP, in the Ti6Al4V group [26]. These findings suggest that titanium alloys not only support bone tissue growth but also actively promote osteogenic differentiation at both the protein and mRNA levels. The increased expression of Runx2, a key transcription factor in osteoblast differentiation and BSP, which is crucial for bone matrix mineralisation, further validates the osteoinductive properties of titanium alloy implants [27].

From a clinical standpoint, the combination of superior mechanical properties and enhanced osteogenic response observed for Ti‐6Al‐4V has several important implications. The higher tensile and compressive strength, together with a lower elastic modulus closer to that of cancellous bone, suggests that Ti‐6Al‐4V implants may better withstand functional loading while minimising stress shielding and preserving peri‐implant bone [28]. In the context of dental implants, this mechanical behaviour is particularly relevant for posterior regions and other high‐load sites, where both fatigue resistance and physiological load transfer are critical for long‐term success [29].

From a manufacturing and translational perspective, it is important to consider whether the proposed Ti‐6Al‐4V implants can be produced at scale in a cost‐effective manner. The alloy composition and mechanical properties investigated in this study are consistent with Ti‐6Al‐4V grades that are already widely used in orthopaedic and dental implants and are covered by international standards such as ASTM F136/F1472 and ISO 5832‐3 [30]. These standards underpin the large‐scale industrial production, quality control and regulatory acceptance of Ti‐6Al‐4V components. The implants in our study were fabricated using conventional machining and surface‐finishing procedures that are compatible with current dental implant manufacturing lines, without requiring specialised or high‐cost processes. Therefore, the transition from experimental implants to industrial‐scale production would primarily rely on existing infrastructure, and the overall manufacturing cost is expected to be comparable to that of conventional titanium implants. This combination of established material standards, compatibility with routine manufacturing routes and improved mechanical and biological performance suggests that the proposed Ti‐6Al‐4V implants are not only clinically promising but also industrially feasible and scalable.

Taken together, the enhanced mechanical properties and superior biological performance of Ti‐6Al‐4V implants suggest that they are particularly suitable for clinical applications where long‐term stability, biocompatibility and osseointegration are critical. The reduced apoptosis and upregulation of osteogenic markers provide strong evidence that Ti‐6Al‐4V promotes faster and more efficient bone healing and integration, making it a promising choice for both load‐bearing and non‐load‐bearing dental implant applications [26]. Nevertheless, several limitations remain. The present study focused on a single murine pre‐osteoblast line and a rat mandibular model; thus, extrapolation to human clinical scenarios should be made with caution. Long‐term in vivo evaluations, including large‐animal models and human clinical data, are needed to confirm the durability and safety of Ti‐6Al‐4V implants. In addition, the influence of specific surface modifications, which have been shown to further enhance osseointegration, was not explored here and warrants future investigation.

## 5 Conclusion

This study demonstrates that Ti‐6Al‐4V implants provide a distinct mechanical and biological advantage over a conventional Ti–Al–V alloy. Mechanically, Ti‐6Al‐4V achieved an ultimate tensile strength of 1200 ± 50 MPa and an elongation at break of 15.2% ± 2.1%, compared with 950 ± 45 MPa and 9.8% ± 1.7% for the control alloy, respectively. The compressive yield and ultimate strengths of Ti‐6Al‐4V (850 ± 40 MPa and 1300 ± 60 MPa) also exceeded those of the conventional alloy (720 ± 35 MPa and 1100 ± 50 MPa), while its elastic modulus (10.8 ± 0.15 GPa) was substantially lower than that of the control (16.2 ± 0.18 GPa), approaching that of cancellous bone and indicating reduced stress‐shielding risk. Biologically, Ti‐6Al‐4V surfaces supported significantly higher cell proliferation and ALP activity in MC3T3‐E1 cells and promoted greater bone formation, lower apoptosis and increased expression of Runx2 and BSP in a rat mandibular model (all *p* < 0.05). Together, these quantitative findings indicate that Ti‐6Al‐4V combines high strength with favourable stiffness and osteogenic support, underscoring its strong potential for load‐bearing and non‐load‐bearing dental and orthopaedic implant applications.

## 6 Future Perspectives

Although the present study demonstrates that Ti‐6Al‐4V implants exhibit superior mechanical properties, biocompatibility and osteogenic potential compared with a conventional Ti–Al–V alloy, further work is required to consolidate and extend these findings. First, future in vitro studies should incorporate human‐derived osteoblasts or mesenchymal stem cells, as well as co‐culture systems that include immune or endothelial cells, to better recapitulate the complex human peri‐implant microenvironment. Second, long‐term in vivo investigations in large‐animal models are needed to evaluate fatigue behaviour, marginal bone maintenance and biological safety under clinically relevant loading conditions. Third, systematic optimisation of surface modifications on the Ti‐6Al‐4V substrate may further enhance osseointegration and soft‐tissue integration. Finally, prospective clinical trials will be essential to validate whether the mechanical and osteogenic advantages observed here translate into improved implant survival, reduced complication rates and better functional outcomes in patients, especially those with compromised bone quality.

## Author Contributions

**Huayan Guo:** conceptualization, investigation, funding acquisition, writing – review and editing, validation, formal analysis, project administration, software, resources, supervision, data curation. **Zhuo Wang:** conceptualization, writing – original draft, writing – review and editing, visualization, validation, methodology, formal analysis, software, data curation, supervision, resources, investigation.

## Funding

The authors have nothing to report.

## Ethics Statement

The ethic approval was reviewed and approved from Shanghai East Hospital, School of Medicine, Tongji University.

## Consent

Informed written consent was obtained from all patients.

## Conflicts of Interest

The authors declare no conflicts of interest.

## Data Availability

The data that support the findings of this study are available from the corresponding author upon reasonable request.
